# Intranasal administration with recombinant vaccine PRVXJ-delgE/gI/TK-S induces strong intestinal mucosal immune responses against PDCoV

**DOI:** 10.1186/s12917-023-03739-5

**Published:** 2023-09-23

**Authors:** Bingzhou Huang, Yao Huang, Lishuang Deng, Tong Xu, Zhijie Jian, Siyuan Lai, Yanru Ai, Ling Zhu, Zhiwen Xu

**Affiliations:** 1 College of Veterinary Medicine, Sichuan Agricultural University, Chengdu, 611130 China; 2 Key Laboratory of Animal Diseases and Human Health of Sichuan Province, Chengdu, 611130 China

**Keywords:** PRV, PDCoV, Spike protein, Recombinant vaccine, Intestinal mucosal immunity

## Abstract

Porcine deltacoronavirus (PDCoV) is a novel coronavirus that causes enteric diseases in pigs leading to substantial financial losses within the industry. The absence of commercial vaccines and limited research on PDCoV vaccines presents significant challenges. Therefore, we evaluated the safety and immunogenicity of recombinant pseudorabies virus (PRV) rPRVXJ-delgE/gI/TK-S through intranasal mucosal immunization in weaned piglets and SPF mice. Results indicated that rPRVXJ-delgE/gI/TK-S safely induced PDCoV S-specific and PRV gB-specific antibodies in piglets, with levels increasing 7 days after immunization. Virus challenge tests demonstrated that rPRVXJ-delgE/gI/TK-S effectively improved piglet survival rates, reduced virus shedding, and alleviated clinical symptoms and pathological damage. Notably, the recombinant virus reduced anti-inflammatory and pro-inflammatory responses by regulating IFN-γ, TNF-α, and IL-1β secretion after infection. Additionally, rPRVXJ-delgE/gI/TK-S colonized target intestinal segments infected with PDCoV, stimulated the secretion of cytokines by MLVS in mice, stimulated sIgA secretion in different intestinal segments of mice, and improved mucosal immune function. HE and AB/PAS staining confirmed a more complete intestinal mucosal barrier and a significant increase in goblet cell numbers after immunization. In conclusion, rPRVXJ-delgE/gI/TK-S exhibits good immunogenicity and safety in mice and piglets, making it a promising candidate vaccine for PDCoV.

## Introduction

Porcine deltacoronavirus (PDCoV) is an emerging porcine enterovirus belonging to the family Coronaviridae and genus Coronaviruses. It can co-infect with enterovirus, such as porcine epidemic diarrhea virus (PEDV), causing severe vomiting, watery diarrhoea, dehydration, and even death in pigs. PDCoV was initially discovered in Hong Kong in 2012 and emerged in Ohio and Indiana in the United States in February 2014 [1, 2]. Subsequently, it spreaded rapidly in the United States and Canada, leading to a global epidemic and causing significant economic losses to the pig industry [3, 4]. A systematic review was conducted to access the prevalence of PDCoV infection in pig population of China, revealing an estimated prevalence of 12.2% [5]. Recent studies have shown that PDCoV can efficiently infect a wide range of cells, including humans, suggesting its potential for rapid and cross-species transmission [6–8]. Consequently, preventing and controlling PDCoV not only has economically significant but also has important for public health security.

The PDCoV genome is approximately 25.4 kb in size and encodes four major structural proteins, including spike (S), envelope (E), membrane (M), and nucleocapsid (N), as well as three non-structural proteins NS6, NS7, and NS7a [8, 9]. The S protein is particularly essential in determining host range, tissue tropism, and virus virulence, and also inducing neutralizing antibodies, making it an ideal target for vaccine development [10, 11]. Currently, no commercial vaccines are available for PDCoV. Research on PDCoV vaccines mainly focuses on inactivated vaccines, which have limitations in protection against homologous viruses [12, 13].

Live vaccines based on recombinant viruses have been shown to provide stronger immune protection and often cause better immune response and duration than inactivated vaccines [14]. Additionally, they have proven to be more effective in resisting homologous virus challenge. [15]. PRV, a recombinant live vaccine vector, has been used in the research and development of a variety of viral vaccines [16]. The TK gene, which is not involved in virus replication, is commonly deleted to reduce the infectivity of the virus to nervous tissues, making it an attractive target for the development of live attenuated vaccines. Moreover, the gE gene serves as an effective marker in distinguishing vaccine strains from wild-type viruses and ought to be deleted in the development of any PRV vaccine [17–20].

In the preliminary experiment, we used CRISPR/Cas gene editing and homologous recombination technology to construct a recombinant virus, PRVXJ-delgE/gl/TK-S, that expresses the PDCoV S protein [21]. The recombinant virus demonstrated good safety and immunogenicity in mice. However, since PDCoV primarily infects the intestine and elicits a strong intestinal mucosal immune response, we further assessed the colonization of the recombinant virus in different intestinal segments of mice and its ability to induce sIgA secretion. Furthermore, it should be noted that,mouse models do not provide a complete representation of the performance of the recombinant virus in pigs. Therefore, we inoculated piglets with the recombinant virus PRV rPRVXJ-delgE/gI/TK-S and evaluated their health status, immune response, and protection against PDCoV infection. It is suggested that PRVXJ-delgE/gl/TK-S is a promising candidate vaccine for the prevention and control of PDCoV infection.

## Materials and methods

### Vaccines, cells and viruses

The rPRVXJ-delgE/gI/TK-S, rPRVXJ-delgE/gI/TK-EGFP and PRV XJ strains were constructed and preserved by Animal Biotechnology Center, College of Veterinary Medicine, Sichuan Agricultural University (Chengdu, China), and proliferated on BHK-21 cells. Each virus stock was titrated by TCID_50_ following the Reed-Muench method on BHK-21 cells. All vaccines were confirmed to be free of bacteria and fungi according to the standard method as described in the Veterinary Phamacopoeia of the People’s Republic of China 2015 Edition, and stored at -80℃ until use.

PDCoV was preserved by Animal Biotechnology Center, College of Veterinary Medicine, Sichuan Agricultural University (Chengdu, China), and proliferated on LLC-PK1 cells.

BHK-21 cells were cultured in DMEM (Gibco, New York, USA) supplemented with 10% newborn bovine serum (NBS) (37℃, 5% CO_2_). LLC-PK1 cells were cultured in DMEM (Gibco, New York, USA) supplemented with 10% NBS (37℃, 5% CO_2_).

### Colonization of recombinant virus rPRVXJ-delgE/gI/TK-S in mice

All the animal experiments were approved by the Laboratory Animal Management Committee of Sichuan Province (Approval Number SYXK2019-187).

Female BALB/c mice aged 6–8 weeks were purchased from Chengdu Dashuo Biotechnology Co., Ltd. (Chengdu, China). A total of 120 mice were randomly divided into two groups (80 mice in the rPRVXJ-delgE/gI/TK-S group and 40 mice in the DMEM group) and inoculated with activated 5 × 10^6^ TCID_50_ rPRVXJ-delgE/gI/TK-S vaccine and 50 µl DMEM by nasal drop immunization. Two weeks later, the booster immunization was administered. All surviving mice were euthanized by an overdose of sodium pentobarbital (National Pharmaceutical Group Corporation, China) dosed by intravenous route and a complete necropsy was performed.

### Immunohistochemistry analysis of mucosal sIgA in mice

In order to analyze the secretion of IgA in different intestinal sections (jejunum, ileum, cecum, colon and rectum) of mice in the experimental and control groups at each time points (1 dpi, 3 dpi, 7 dpi and 14 dpi), 4% paraformaldehyde fixed intestinal samples were sent to Wuhan Servicebio for immunohistochemical analysis. Goat anti-mouse sIgA (Abcam, Cambridge, UK) was used as primary antibody, goat anti-mouse IgG (Servicebio, Wuhan, China) as secondary antibody. Image-Pro Plus 6.0 software was employed to calculate the percentage of positive cells.

### Test of vaccine safety of rPRVXJ-delgE/gI/TK-S

Nine 3-week-old healthy piglets were purchased from Wanjiahao pig farms without PDCoV and PRV for immunization safety test. Prior to vaccination, all pigs were tested negative for PRV antibodies. The pigs were randomly divided into three groups, with three pigs in each group. Group 1 was intramuscularly vaccinated with 2 mL inactivated vaccine prepared from rPRVXJ-delgE/gI/TK-S at a dose of 10^7^ TCID_50_. Group 2 was intramuscularly vaccinated with 2 mL inactivated vaccine prepared from rPRVXJ-delgE/gI/TK-S at a dose of 10^6^ TCID_50_. Group 3 was intramuscularly inoculated with 2 mL DMEM as a negative control. Each group was housed in separate rooms at an ambient temperature of 20–25℃. Clinical signs were monitored daily for 14 days. Serum samples were collected for PRV gB and gE antibodies test at 14 days P.I.

After 14 days, all surviving pigs were euthanized and various tissue samples (including brain, heart, liver, spleen, lung, kidney, lymph nodes and intestinal) were collected and subjected to pathological examinations [22].

### Test of vaccine efficacy of rPRVXJ-delgE/gI/TK-S

Nine 3-week-old healthy piglets purchased from a PDCoV-free pig farm were used to test the immunogenicity of the vaccine. Prior to vaccination, all pigs tested negative for PRV antibodies, viral DNA and RNA by the PRV antibody detection kit (ID.VET, Montpellier, France), the PCR and RT-PCR, respectively. The pigs were randomly divided into three groups (A-F), with three pigs in each group. Groups A and B were intranasally vaccinated with 2 mL vaccine prepared from rPRVXJ-delgE/gI/TK-S at a dose of 10^6^ TCID_50_. Groups C and D were also intranasally vaccinated with 2 mL vaccine prepared from rPRVXJ-delgE/gI/TK-EGFP at a dose of 10^6^ TCID_50_. Groups E and F were intranasally inoculated with 2 mL DMEM as a negative control (Table 1). Each group was housed separately in a temperature-controlled room maintained at 20–25. Booster immunizations were given at the same dose by intramuscular injection 4 weeks after the primary immunization. Blood samples were collected at 2, 4, 6, and 8 weeks after initial immunization. Four weeks after vaccination, the piglets in groups A, C and E were challenged intranasally with 10^5^ TCID_50_ of PRV XJ, and the piglets in groups B, D and F were challenged with PDCoV by intragastric administration. After the challenge, clinical symptoms and rectal temperatures were monitored, and virus excretion was determined by collecting anal swabs at 2, 3, 5, 7-days and 14-days after the challenge (dpc). The challenged piglets were blood sampled at 7 dpc and 14 dpc.

After 14 dpc, all surviving pigs were euthanized by an overdose of sodium pentobarbital (National Pharmaceutical Group Corporation, China) dosed by intravenous route and different tissue samples (including brain, heart, liver, spleen, lung, kidney, lymph nodes and intestinal) were collected and subjected to pathological examinations.

**Table 1:** The immunity and challenge of each group in the piglet challenge experiment

Group	Vaccines for immunization	The challenged virus
A	2mL rPRVXJ-delgE/gI/TK-S	PRV
B	2mL rPRVXJ-delgE/gI/TK-S	PDCoV
C	2mL rPRVXJ-delgE/gI/TK-EGFP	PRV
D	2mL rPRVXJ-delgE/gI/TK-EGFP	PDCoV
E	2mL DMEM	PRV
F	2mL DMEM	PDCoV

### Neutralizing antibody assay

To assess the neutralizing antibody titer of rPRVXJ-delgE/gI/TK-S, the antigen-specific and neutralizing antibodies were detected using a virus-specific neutralizing antibody (VNA) assay. Dilutions of serum were co-incubated with the virus (0.2–200 TCID_50_) for 1 h at 37℃ prior to the addition of LLC-PK1 cells in DMEM containing 1% NBS and 1% PS. After 3 days of incubation at 37℃ and 5% CO_2_, the neutralization was analyzed by observing cytopathic effects (CPEs) under a microscope.

### ELISA for antibodies

To evaluate the specific antibody and cytokines response to rPRVXJ-delgE/gI/TK-S, We measured the levels of specific antibodies to PRV-gB, PRV-gE, and PDCoV-S and the levels of cytokines IFN-γ, IL-1β, IL-6, IL-8, IL-10, TNF-α, and IL-2 in the blood of piglets with ELISA. PRV-gB and PRV-gE antibody ELISA kit were purchased from IDEXX (Maine, USA). ELISA kits for cytokine detection were purchased from Thermo Fisher Scientific (Massachusetts, USA). All operations were carried out according to the instructions of commercial ELISA kit. PDCoV anti-S antibody was detected by PDCoV-S antibody ELISA assays constructed in our lab [18].

### Viral load was detected by real-time quantitative RT-PCR

To evaluate the protective effect of the recombinant virus rPRVXJ-delgE/gI/TK-S, viral loads of PRV and PDCoV in blood and anal swabs were measured. The viral DNA/RNA was purified from a 1 g tissue sample using the Magnetic Pathogen DNA/RNA Kit (Vazyme, Nanjing, China) according to the manufacturer’s instructions. The qPCR test was conducted at a total volume of 20.0 µL containing 10.0 µL 2× TaqMan Fast Advanced Master Mix (ThermoFisher, Massachusetts, USA), 2.0 µL DNA, 6.5 µL sterilized H_2_O, and 0.5 µL each of the primers (final primer concentration 0.5 mM). The reaction was heated to 95℃ for 1 min, followed by 40 cycles of 95℃ for 5 s, and 60℃ for 20 s (fluorescence captured). TaqMan real-time PCR was carried out using the QuantStudio 5 real-time PCR instrument (ThermoFisher, Massachusetts, America). According to the CT value of each tissue sample, the original copies were calculated by the standard curve constructed in the previous study [15, 23] and converted into copies of the virus load in each gram of tissue with multiples (25×). The copy numbers of each tissue sample were expressed as log10 copies per gram of tissue sample.

### Hematoxylin - eosin staining

All tissue of pigs was fixed with 4% paraformaldehyde and sent to Wuhan Servicebio for pathological examination.

### AB/PAS (alcian blue/periodic acid Schiff) staining

All intestinal tissues of pigs were fixed with 4% paraformaldehyde and sent to Wuhan Servicebio for AB/PAS staining.

## Result

### Immunohistochemical analysis of virus colonization in intestinal mucosa of mice

**Fig. 1:**
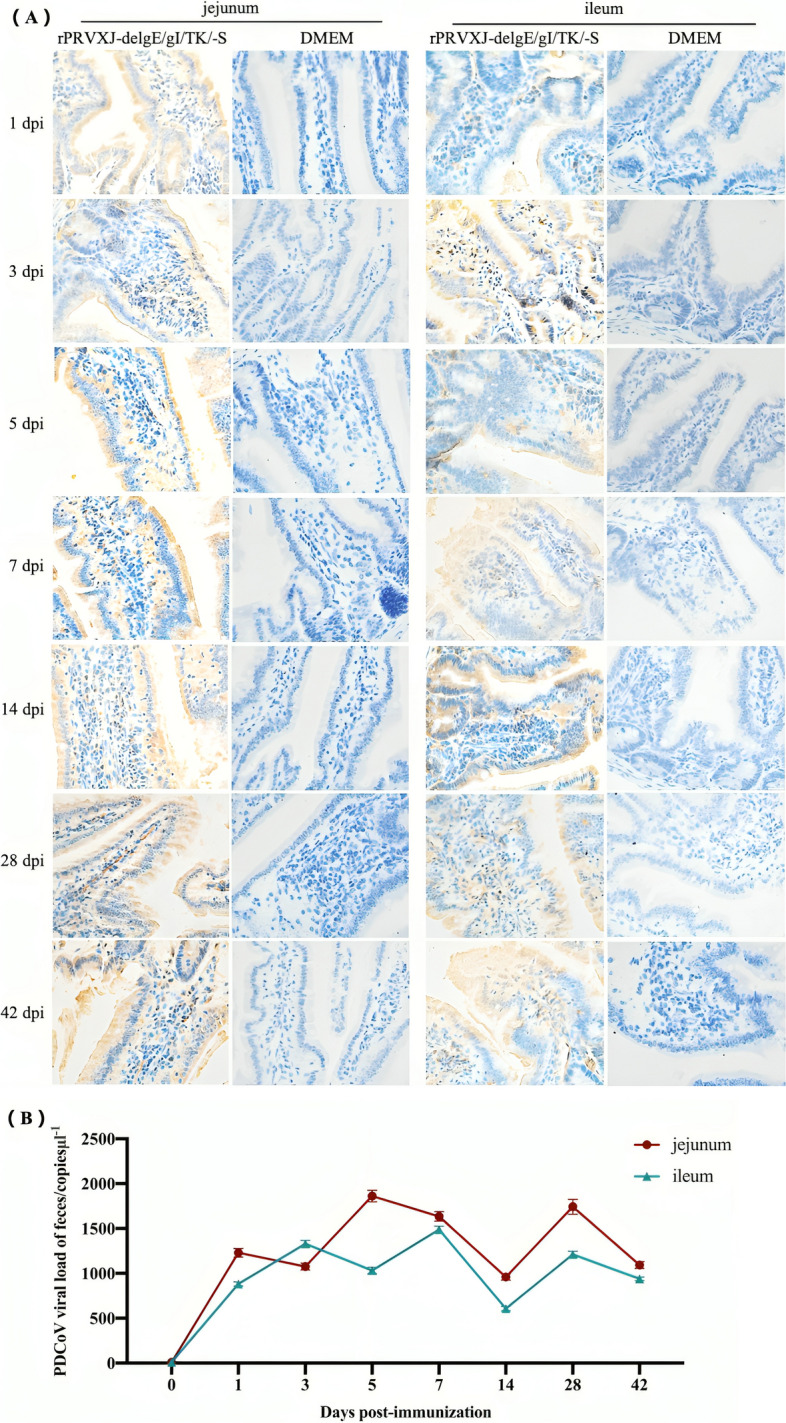
Colonization of recombinant virus rPRVXJ-delgE/gI/TK-S of mice. **A** The colonization of recombinant virus rPRVXJ-delgE/gI/TK-S in jejunum and ileum of mice was analyzed by immunohistochemistry using PDCoV S protein polyclonal antibody as primary antibody and goat anti-mouse IgG as secondary antibody. (immunohistochemistry; × 400) (**B**) Quantitative analysis of rPRVXJ-delgE/gI/TK-S tissue in jejunum and ileum

In the preliminary study, we have measured the basic indicators such as mortality, neutralizing antibody level, and specific antibody level of mice immunized with rPRVXJ-delgE/gI/TK-S. These results have been published [18]. To further verify the protective ability of rPRVXJ-delgE/gI/TK-S on the intestinal mucosa, this study analyzed the colonization of rPRVXJ-delgE/gI/TK-S in the mouse intestine by immunohistochemistry, and found that rPRVXJ-delgE/gI/TK-S could colonize the jejunum and ileum of mice, while rPRVXJ-delgE/gI/TK-S was not detected in the DMEM group mice (Fig. 1A). Moreover, the recombinant virus rPRVXJ-delgE/gI/TK-S was detected in the jejunum and ileum of the experimental mice at all time points (Fig. 1B).

### Immunohistochemical analysis of sIgA in intestinal mucosa of mice

**Fig. 2:**
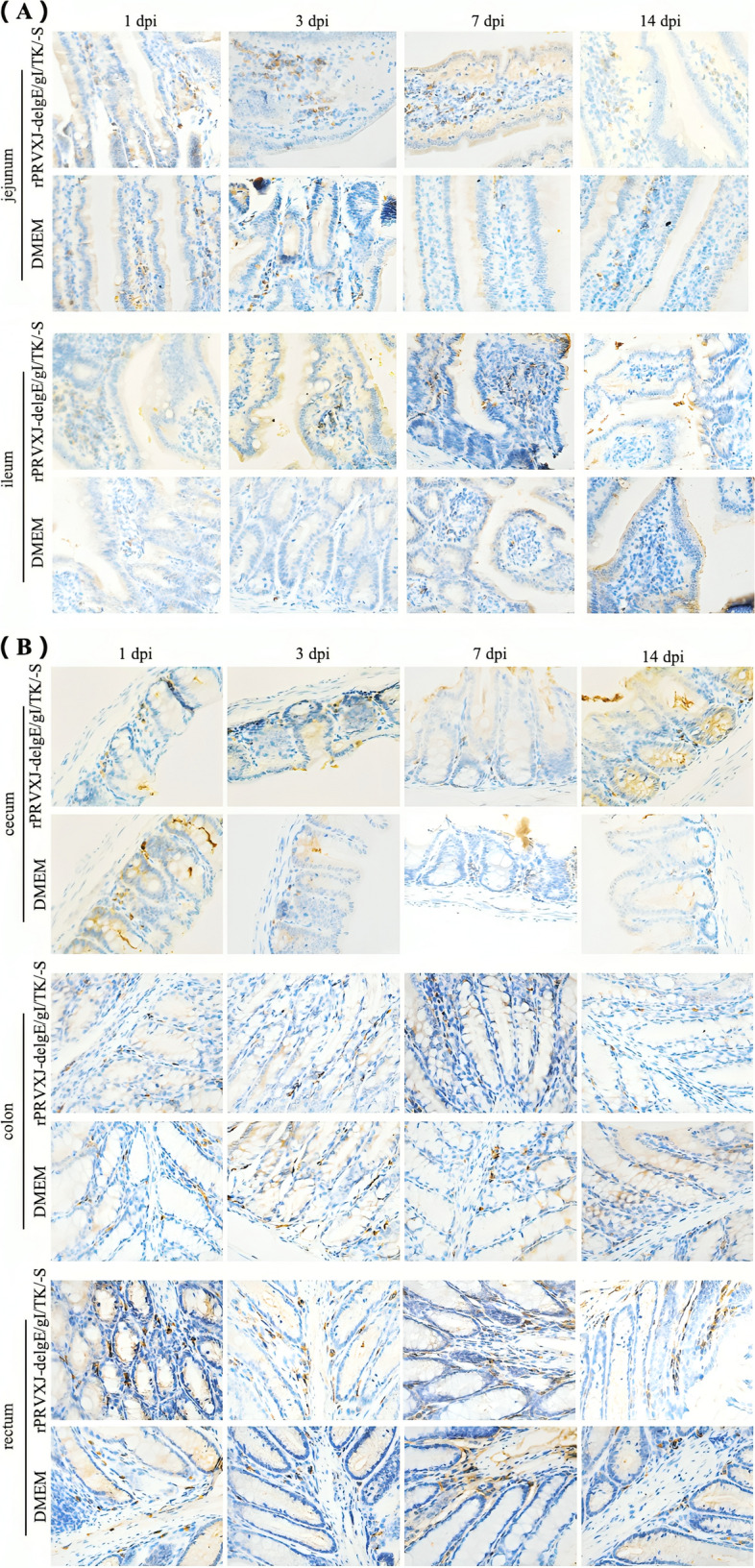
Immunohistochemical analysis of sIgA in intestinal mucosa. **A**, **B** The secretion of IgA in the mucosa of jejunum, ileum, cecum, colon and rectum of mice at 1dpi, 3dpi, 7dpi and 14dpi was analyzed by immunohistochemistry (goat anti-mouse sIgA was the primary antibody and goat Anti-mouse IgG was the secondary antibody). (immunohistochemistry; × 400)

**Fig. 3:**
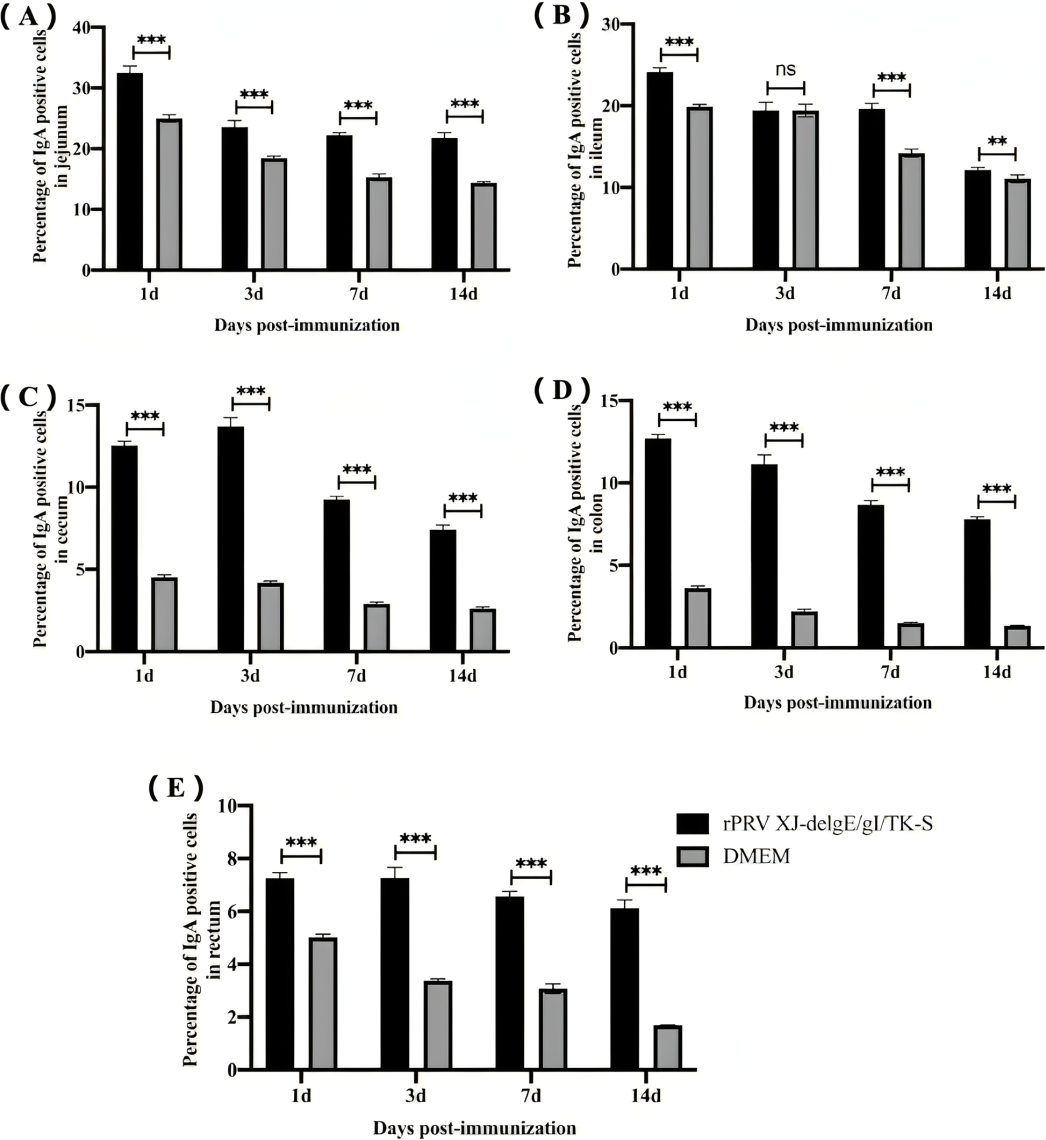
The proportion of sIgA positive cells in each intestinal segment. **A**-**E** The proportion of IgA positive cells in jejunum, ileum, cecum, colon and rectum (1dpi, 3dpi, 7dpi and 14dpi) was calculated by Image Pro Plus 6.0 software. (immunohistochemistry; × 200) Significant difference was indicated by “***” (*P* < 0.001); Extremely significant difference was indicated by “**” (*P* < 0.01); Significant difference was indicated by “*” (*P* < 0.05); No significant difference was indicated by “ns” (*P* > 0.05)

sIgA is the most critical immunoglobulin in the intestinal mucosa. The study determined the mucosal content of sIgA by performing an immunohistochemistry analysis (Fig. 2). The rPRVXJ-delgE/gI/TK-S group showed a higher percentage of sIgA positive cells in the intestinal epithelium compared to the DMEM group (Fig. 3).

### The secretion of IL-4 and IFN-γ by MLN after immunization

**Fig. 4:**
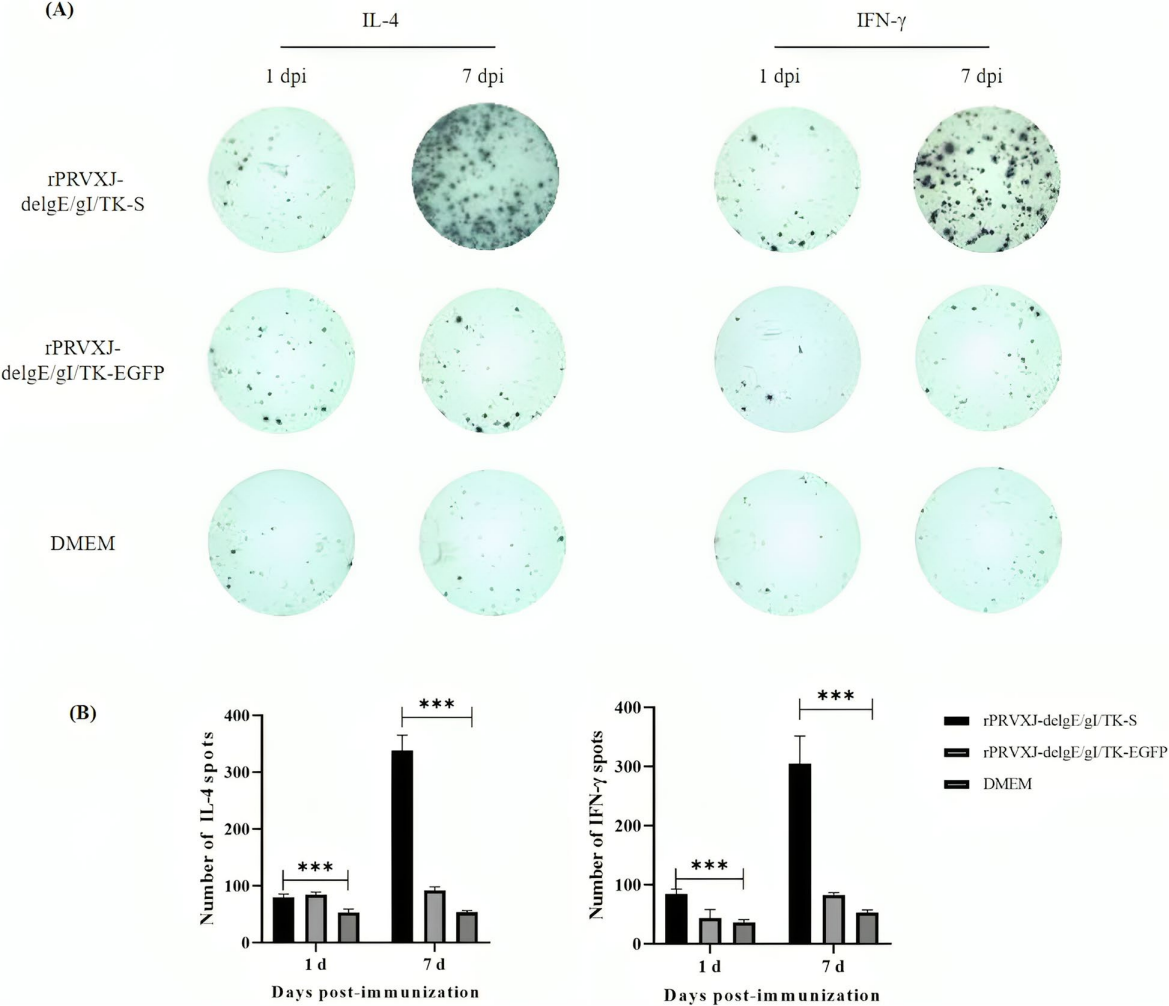
The secretion of IL-4 and IFN-γ by MLN after immunization was detected by ELISPOT. **A** Typical images of ELISPOT Wells of intestinal cells in mice. **B** Spot counts in ELISPOT Wells of MLV in mice. Significant difference was indicated by “***” (*P* < 0.001); Extremely significant difference was indicated by “**” (*P* < 0.01); Significant difference was indicated by “*” (*P* < 0.05); No significant difference was indicated by “ns” (*P* > 0.05)

To further analyze the impact of the rPRVXJ-delgE/gI/TK-S on the secretion of IL-4 and IFN-γ by mesenteric lymph node (MLN) cells in mice, enzyme-linked immunospot (ELISPOT) assay was performed on MLN cells from mice on days 1 and 7 after the second immunization. We measured the secretion and production of IL-4 and IFN-γ by MLN cells after immunisation. The findings indicate that MLN cells, stimulated by rPRVXJ-delgE/gI/TK-S, produced elevated levels of IL-4 and IFN-γ, significantly differing from the control group (*P* < 0.001) (Fig. 4A and B).

### The secretion of sIgA in the intestinal tract of mice stimulated by PDCOV-S

**Fig. 5:**
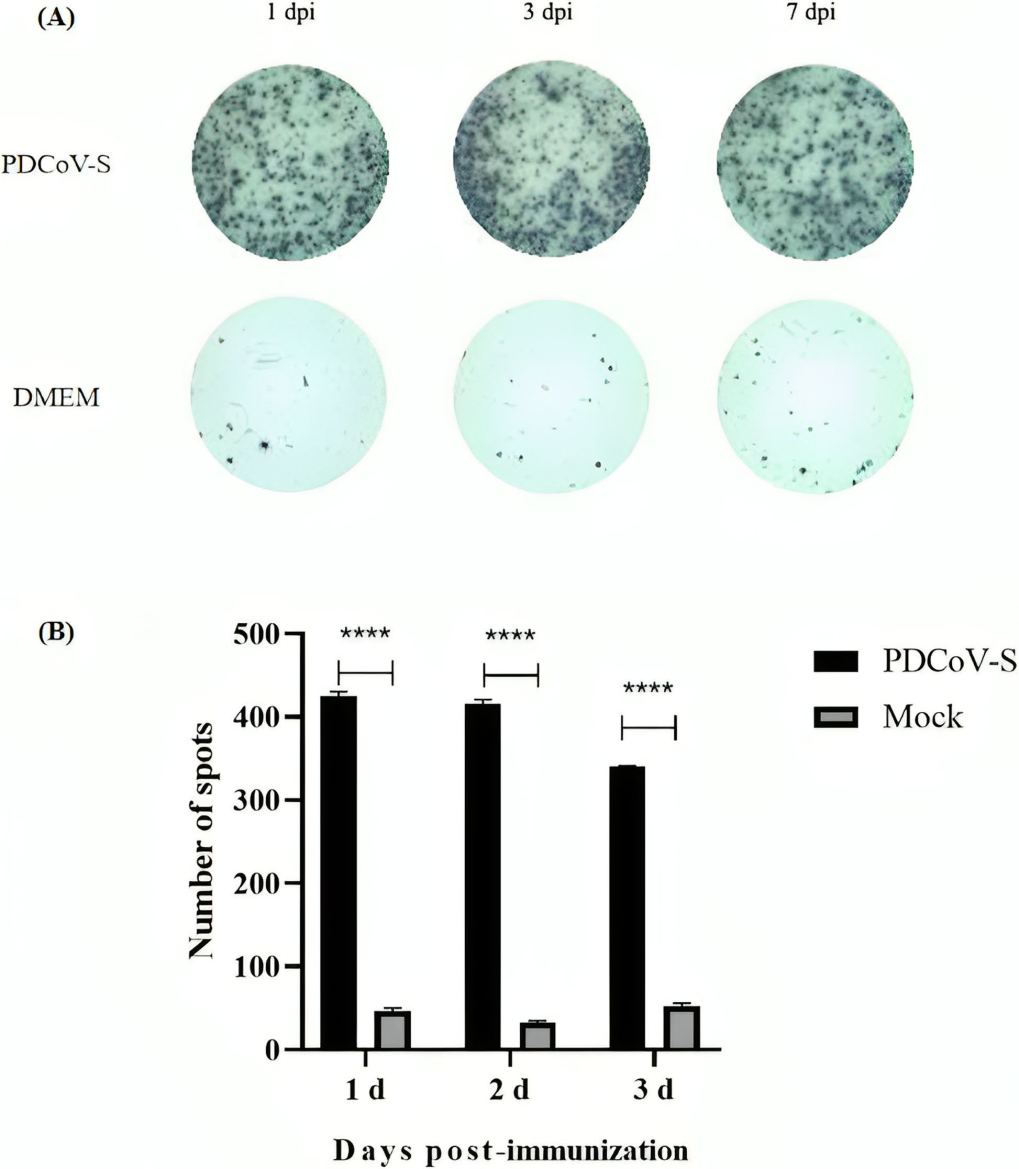
The secretion of sIgA in the intestinal tract of mice stimulated by PDCOV-S was detected by ELISPOT. **A** Typical images of sIgA-ELISPOT Wells of intestinal cells in mice. **B** Spot counts in sIgA-ELISPOT Wells of intestinal cells in mice. Significant difference was indicated by “***” (*P* < 0.001); Extremely significant difference was indicated by “**” (*P* < 0.01); Significant difference was indicated by “*” (*P* < 0.05); No significant difference was indicated by “ns” (*P* > 0.05)

To further analyze the stimulatory effect of PDCOV-S on the secretion of secretory IgA (sIgA) in the intestinal mucosa of mice, enzyme-linked immunospot (ELISPOT) assay was performed on intestinal cells from mice at 1, 3, and 7 days after the second immunization. The ability of intestinal mucosal secretion to produce sIgA after immunization was examined. The results demonstrated that PDCOV-S stimulated mice to produce a higher level of sIgA, which was significantly different from the control group (*P* < 0.0001) (Fig. 5).

### rPRVXJ-delgE/gI/TK-S was avirulent to piglets

**Fig. 6:**
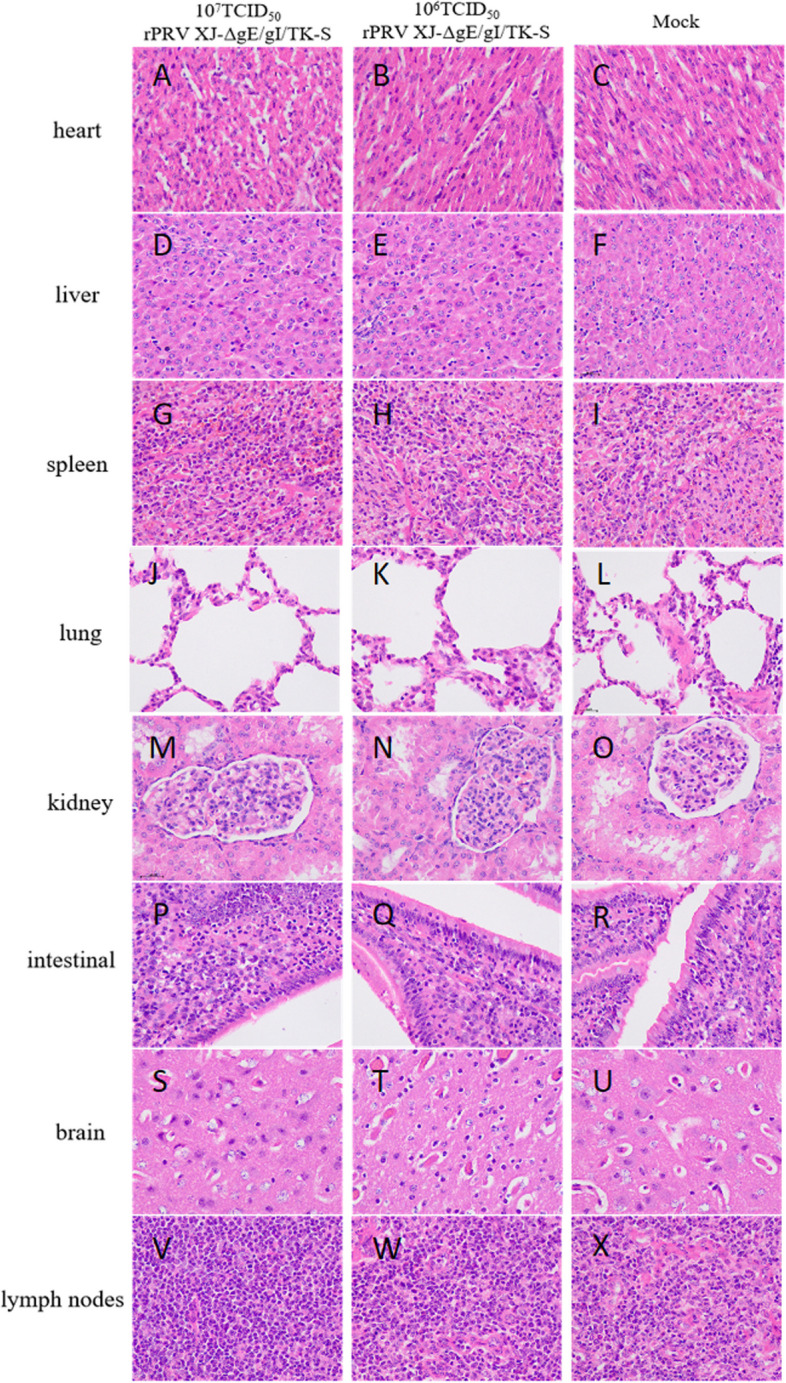
Histopathological analysis of piglets after immunization. **A**, **D**, **G**, **J**, **M**, **P**, **S**, **V** 10^7^ TCID_50_ recombinant virus rPRVXJ-delgE/gI/TK-S immunized piglets; (B,E,H,K,N,Q,T,W): 10^6^ TCID_50_ recombinant virus rPRVXJ-delgE/gI/TK-S immunized piglets; **C**, **F**, **I**, **L**, **O**, **R**, **U**, **X** Mock piglets; **A**-**C** Heart; **D**-**F **Liver; **G**-**I** Spleen; **J**-**L** Lung; **M**-**O** Kidney; **P**-**R** Intestinal; **S**-**U** Brain; **V**-**X **Lymph nodes

To determine the pathogenicity of rPRVXJ-delgE/gI/TK-S in piglets, groups 1 and 2 were inoculated with 10^7^ TCID_50_ or 10^6^ TCID50 of the viurs, respectively. All piglets survived throughout the experiment without any clinical symptoms. All surviving piglets were euthanized at 14 days post inoculation (dpi) and necropsied. No visible gross pathological lesions were identified in the inoculated piglets’ organs. Histopathological examination showed that the piglets of all groups exhibited almost no pathological lesions (Fig. 6). The results indicated that rPRVXJ-delgE/gI/TK-S was avirulent to sucking piglets.

### Immunogenicity of the rPRVXJ-delgE/gI/TK-S in piglets

**Fig. 7:**
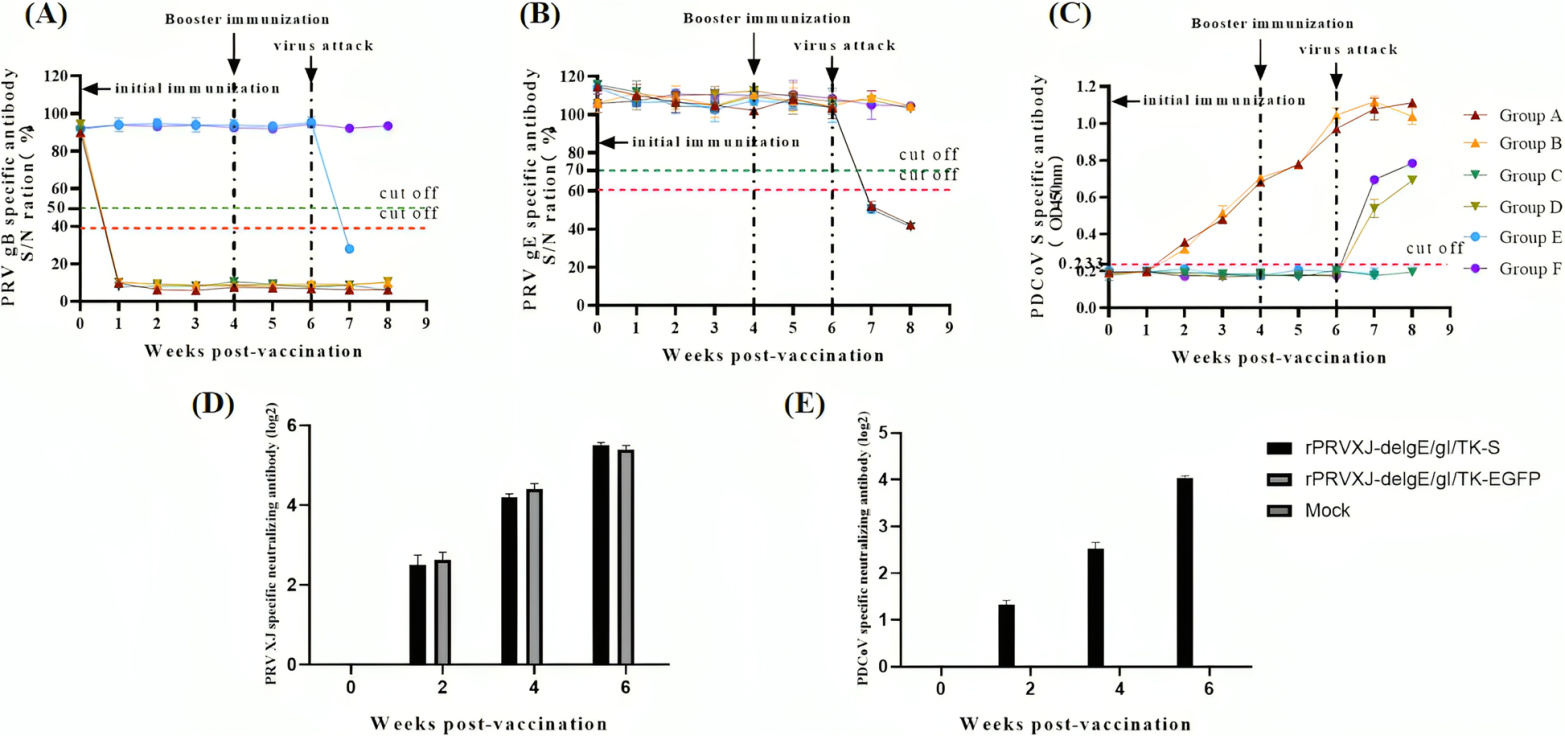
Antibody detection. **A** gB-specific antibodies. gB-specific antibodies were detected by ID.VET blocking ELISA kit. S/N% less than or equal to 40% is positive; S/N% greater than 40% and less than or equal to 50% is suspected; S/N% greater than 50% is negative. **B** gE-specific antibodies. The gE-specific antibodies were detected by ID.VET blocking ELISA kit. S/N% less than or equal to 60% is positive; S/N% greater than 60% and less than or equal to 70% is suspected; S/N% greater than 70% is negative. **C** S-specific antibodies. The S-specific antibody was detected by indirect ELISA method of PDCoV S protein polypeptide antibody constructed in laboratory. OD_450nm_ greater than or equal to 0.233 was positive; OD_450nm_ less than 0.233 is negative. **D** Detection results of anti-PRV XJ-neutralizing antibody titer in the serum of immunized piglets. **E** Detection results of anti-PDCoV-neutralizing antibody titer in the serum of immunized piglets

To monitor PRV gB/gE-specific antibody responses and PDCoV S-specific antibody responses, the serum samples were collected weekly after vaccination. The antibodies to PRV were detected with ELISA. The results indicated that the PRV gB-specific antibody and PDCoV S-specific antibody could be detected at 7 days post-inoculation in group A and B (Fig. 7A-C). Antibody levels remained stable. The PRV gB-specific antibody also could be detected in group C and D at 7 days post-inoculation (Fig. 7A-C).

### Neutralizing antibody assay

The VNA titers against PRV in the rPRVXJ-delgE/gI/TK-S vaccinated group were similar to those in the rPRVXJ-delgE/gI/TK-EGFP vaccinated group (Fig. 7D), and that the VNA titers against PDCOV reached the level required for the vaccine (Fig. 7E).

### Clinical signs post-challenge

**Fig. 8:**
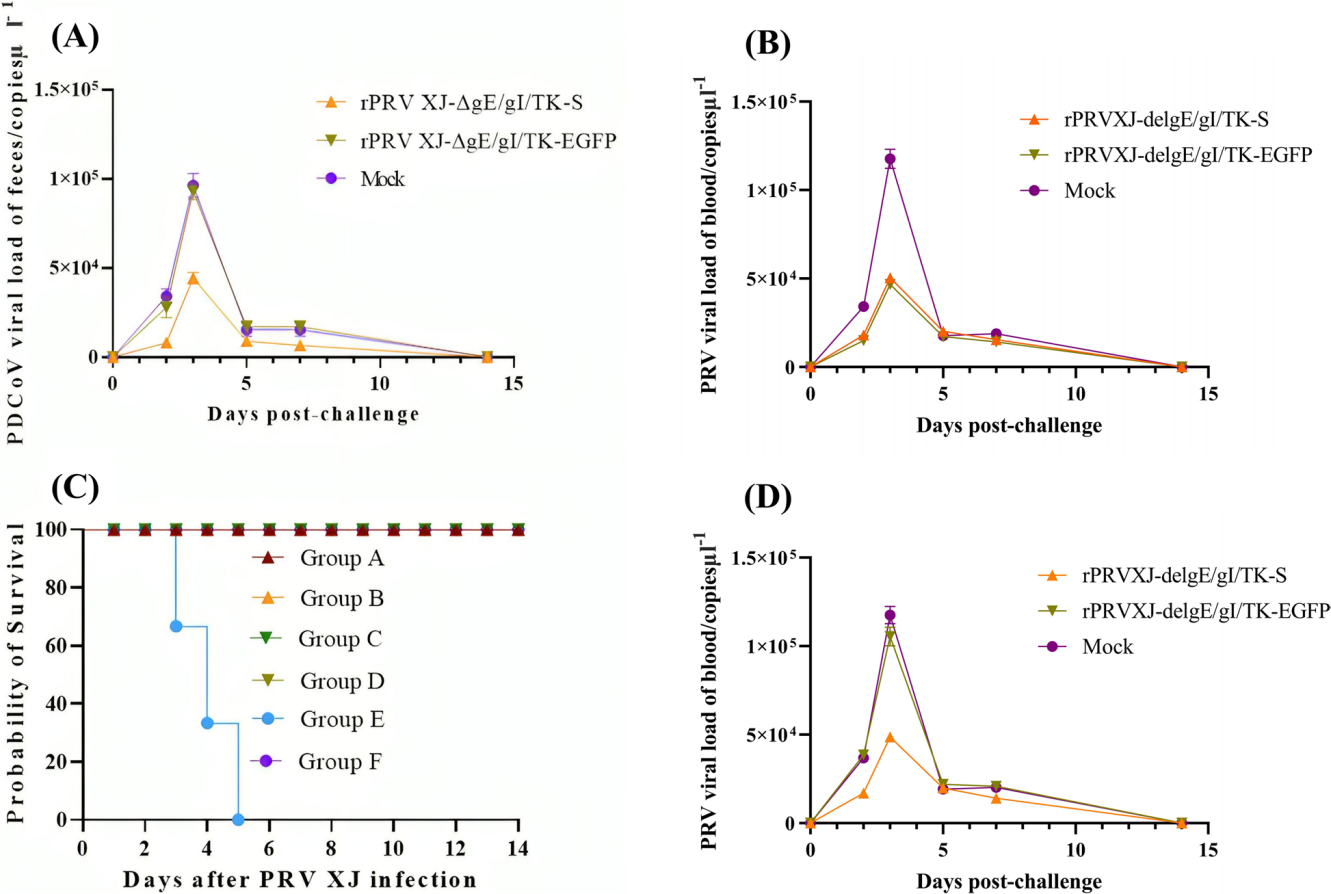
Survival rate and the detoxification of piglets after Challenge. **A** PDCoV viral load of feces in different groups; **B** PRV viral load of blood in different groups; **C** survival rate of piglets in different groups; **D** PDCoV viral load of blood in different groups

Six weeks after vaccination, groups A, C and E were challenged with PRV, while groups B, D and F were challenged with PDCoV. All piglets in group E exhibited typical symptoms of PR, including high fever, depression, anorexia, respiratory distress, vomiting, trembling, and ataxia. On day 5, all piglets in group E died (Fig. 8A). Piglets in group D and F showed clinical symptoms, such as low fever and diarrhea.

### Viruses excretion

In the PDCoV challenge assay, detoxification was detectable in all piglets immunized with rPRVXJ-delgE/gI/TK-S at 2 dpc, with maximum detoxification at 3 dpc. They shed significantly less virus than the rest of the group. In the PDCoV challenge assay, detoxification was detectable at 2 dpc in piglets immunized with rPRVXJ-delgE/gI/TK-S and rPRVXJ-delgE/gI/TK-EGFP, with maximum detoxification at 3 dpc. In contrast, control piglets shed significantly more virus. Mortality statistics verified this result, with piglets only died in the control group (Fig. 8).

### Pathological examination

**Fig. 9:**
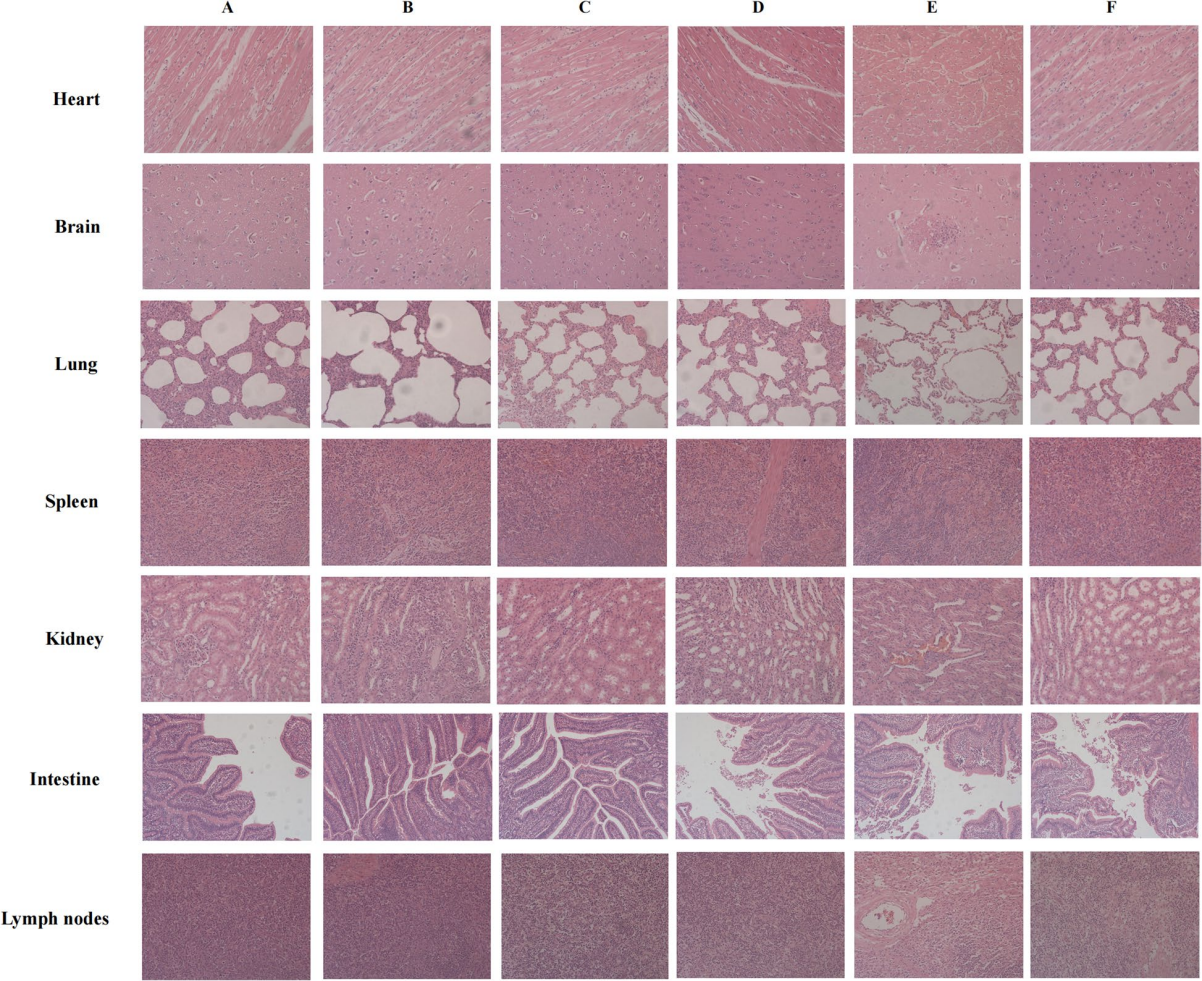
Histopathological analysis of heart, brain, lung, spleen, kidney, intestine, and lymph nodes of piglets in each group after immunization

The piglets in groups A, B and C almost had no pathological lesions, whereas those in group E displayed substantial tissue hemorrhage. Histopathological examination revealed severe lung hemorrhage and congestion, and hemorrhage and inflammatory cell infiltration in the liver, kidney and spleen of the piglets in group E. Shortening and shedding of intestinal villi, mucosal congestion, degeneration and necrosis of mucosal epithelium, and necrosis of small intestinal glands of the piglets in groups D and F (Fig. 9).

### Cytokine detection

**Fig. 10:**
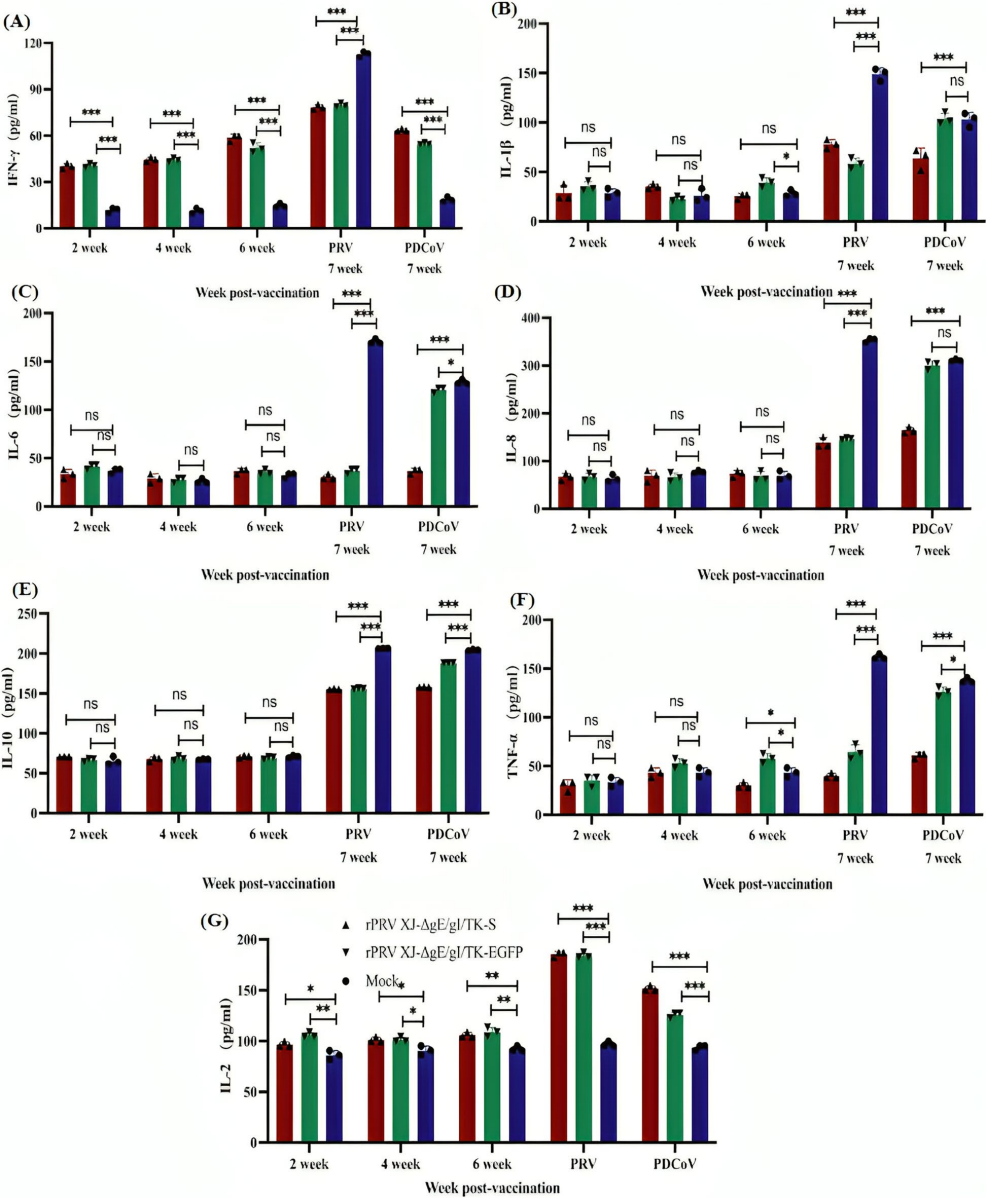
The expression levels of several cytokines in the serum of vaccinated piglets were detected by ELISA. **A** Serum IL-γ was detected by ELISA; **B** Serum IL-1β was detected by ELISA; **C** Serum IL-6 was detected by ELISA; **D** Serum IL-8 was detected by ELISA; **E** Serum IL-10 was detected by ELISA; **F** Serum TNF-α was detected by ELISA; **G** Serum IL-2 was detected by ELISA. Significant difference was indicated by “***” (*P* < 0.001); Extremely significant difference was indicated by “**” (*P* < 0.01); Significant difference was indicated by “*” (*P* < 0.05); No significant difference was indicated by “ns” (*P* > 0.05)

Cytokines play a crucial role in immune responses, reflecting not only immune responses but also the body’s inflammatory response. Serum levels of IL-2, IFN-γ, TNF-α, IL-1β, IL-6, IL-8, and IL-10 were detected at 2, 4, and 6 weeks following immunization and after PDCoV and PRV challenge. At 2, 4, and 6 weeks post-immunization, serum levels of IFN-γ and IL-2 in piglets immunized with rPRVXJ-delgE/gI/TK-S and rPRVXJ-delgE/gI/TK-EGFP increased significantly (***P < 0.001). However, no statistically significant change in serum levels of cytokines IL-2, TNF-α, IL-1β, IL-8, and IL-10 were observed in each group (P > 0.05) (Fig. 10).

After PRV and PDCoV challenge, serum levels of IFN-γ, TNF-α, IL-1β, IL-8, IL-10, and IL-2 in experimental pigs from the rPRVXJ-delgE/gI/TK-S group, rPRVXJ-delgE/g/TK-EGFP group and control group all increased significantly. However, compared to the control group, except for higher levels of IL-2, levels of other detected cytokines were lower with a significant difference (***P < 0.001) (Fig. 10).

### AB/PAS staining

**Fig. 11:**
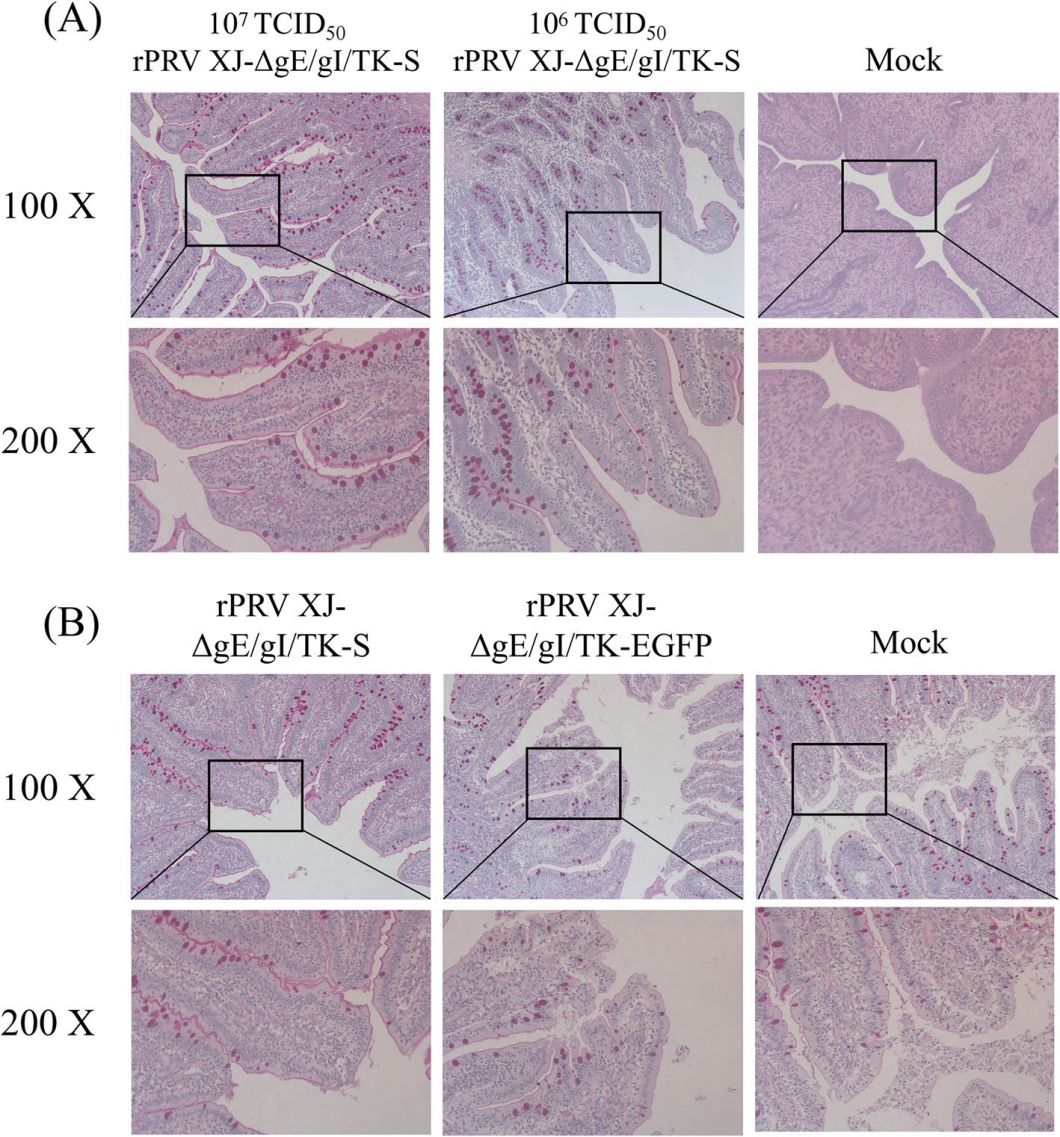
AB/PAS staining analysis of goblet cells in the intestine of piglets. **A** AB/PAS staining of piglets after immunization with different doses of rPRVXJ-delgE/gI/TK-S; **B** AB/PAS staining of piglets from different groups after PDCoV challenge

In a safety trial, the number of intestinal goblet cells in piglets was increased after immunization of piglets with recombinant virus (Fig. 11A). In the challenge protection test, the number of intestinal goblet cells in the immunized piglets was higher than the control group (Fig. 11B).

## Discussion

PDCoV is a novel coronavirus that has emerged in recent years and causes severe diarrhea in piglets. The development of recombinant vaccines is a major focus of current research [24]. Recombinant vaccines are a hot spot in the research and development of new vaccines. These vaccines use the weak virus as a vector, inserting immunodominant antigen genes of other viruses, and highly expressing multiple viral antigens in host cells at the same time. PRV is an ideal vector for recombinant vaccines. Extensive research has involved the insertion of foreign antigen genes into the non-essential genes of gE, gI, and TK deletion PRV strains to construct recombinant vaccines, which have demonstrated the ability to provide multiple protection against PRV and other pathogens [25, 26]. In a previous study, a recombinant PRV rPRVXJ-delgE/gI/TK-S that expresses PDCoV was developed. Its safety and immunogenicity was tested in mice. However, PDCoV causes severe intestinal mucosal damage. Therefore, an examination of the vaccine’s protective effect on the mucosal immune barrier is necessary to determine its efficacy against PDCoV infection. Moreover, we need to verify the safety, immunogenicity and protection of PRV XJ-delgE/gI/TK-S against PRV and PDCoV infection in pigs, which are the target species for the vaccine.

Safety is the primary consideration for any vaccine [27]. To assess the safety of the recombinant vaccine PRV XJ-delgE/gI/TK-S, a pathological analysis was conducted. Results indicate no damage to different tissues and organs in the piglets. Furthermore, immunization did not lead to the production of PRV gE antibodies, indicating the vaccine is safe. The vaccine demonstrated good immunogenicity, generating PRV gB-specific antibodies and PDCoV S-specific antibodies within a week of initial immunization. Additionally, the vaccine significantly reduced the viral load of PDCoV in piglets, indicating its efficacy in providing protection against PDCoV.

To verify the protective efficacy of the vaccine, we conducted PRV and PDCoV challenge tests on piglets immunized with PRV XJ-delgE/gI/TK-S, triple-deficient strain PRV XJ-delgE/gI/TK, and negative control. The piglets vaccinated with the recombinant vaccine and the triple-deficient strain demonstrated effective protection against PRV, as indicated by lower mortality rates, less severe clinical symptoms, and tissue lesions post-challenge. In the PDCoV challenge test, the recombinant vaccine was able to control the viral load.

We also measured cytokine levels, such as IL-2 and IFN-γ in piglet serum 14–28 days post-immunization. The recombinant strains significantly upregulated Th1-type cytokine expression, ,including IFN-γ and IL-2, compared to the control group. This indicating that the vaccine improves the body’s adaptive immune response. This upregulation of IFN-γ expression is crutial in evaluating the efficacy of the vaccine [28]. Furthermore, an increase in IL-2 levels promotes lymphocyte growth, proliferation, and differentiation, playing an essential role in the body’s immune response to viral infections [29]. After the PRV challenge, it was observed that, apart from IL-2 levels, the increase in IFN-γ, TNF-α, IL-1β, IL-8, and IL-10 in the serum of piglets in both the rPRV XJ-ΔgE/gI/TK-EGFP group and the rPRV XJ-ΔgE/gI/TK-S group was not as significant as that in the control group. This trend persisted only in piglets in the rPRV XJ-ΔgE/gI/TK-S group after the PDCoV challenge. The findings suggest that the recombinant vaccine can effectively alleviate the anti-inflammatory and pro-inflammatory responses of piglets after PRV and PDCoV infection, thereby reducing tissue damage caused by viruses. It is noteworthy that IL-2 can also enhance sIgA secretion in the intestine to alleviate the inflammation-induced increase in intestinal mucosal permeability and support the weakening of intestinal mucosal integrity, which is critical in PDCoV infection [30].

PDCoV primarily infects the epithelial surface of the intestinal mucosa, while PRV has been found to negatively impact the intestinal barrier, particularly by inhibiting sIgA secretion in the ileum of piglets [31–33]. The mucosal immune system, particularly the intestinal mucosal immune barrier, is a critical first line of defense against pathogenic microorganisms [34, 35]. Research has shown that the destruction of the intestinal mucosal barrier can lead to an imbalance of Th1, Th2, Th17, and Treg-related cytokine levels, affecting IgE, IgA, and other immunoglobulin levels in the body, resulting in abnormal immunity [36, 37]. sIgA is the most critical immunoglobulin and marker for the mucosal immune response in the intestinal mucosa, and plays a key role in mediating mucosal immune response against enteric pathogens [38]. In this study, we performed immunohistochemical analysis of the intestines of mice immunized with the recombinant vaccine. The results showed that sIgA secretion was earlier than the production of specific antibodies. From the first day of immunization, the percentage of sIgA-positive cells in different intestinal segments of mice vaccinated with the recombinant vaccine was significantly higher than that of the control group. On the 28th day of immunization, the percentage of sIgA-positive cells in the ileum of mice had risen by approximately 50%, while the proportion in the colon increased by more than 6 times. This suggests that sIgA performs a crucial function in initial intestinal mucosal immunity, and is the first immune barrier to prevent pathogen adhesion and colonization in the intestinal mucosa.

To further evaluate the protective effect of the recombinant vaccine PRV XJ-delgE/gI/TK-S on the intestinal mucosal immune barrier, we performed pathological examination, AB/PAS test and immunohistochemical analysis to assess the intestinal health status of piglets after being challenged. The results were consistent with our previous experiments in mice, indicating that PRV XJ-delgE/gI/TK-S could significantly reduce the damage of intestinal villi, mucosal epithelium and intestinal glands caused by PDCoV and PRV in piglets. Previous studies have shown that intestinal epithelial cells are mainly composed of absorptive cells, goblet cells, paneth cells, and endocrine cells [39]. These cells are constantly renewed to ensure the integrity of the intestinal epithelium and maintain the normal function of the intestinal mucosal barrier [40]. Goblet cells are the major secretory cells of the GI tract. They secrete mucin particles, which are mixed with water to form mucus, which covers the surface of the intestinal mucosa to form a protective layer. Therefore, the number and morphology of goblet cells and the expression of mucin can reflect the condition of the intestinal mucosa. AB/PAS experiments showed that PRV XJ-delgE/gI/TK-S could stimulate the secretion of mucus from intestinal goblet cells and protect the intestinal mucosal immune barrier of piglets. These results suggest that the recombinant PRV XJ-delgE/gI/TK-S vaccine can effectively maintain the integrity and homeostasis of intestinal mucosa and environment, thereby better preventing and repairing intestinal barrier damage caused by PDCoV and PRV.

## Data Availability

The raw data is available from the corresponding author upon reasonable request.
